# Validation of a Gastric-Juice-Analysis-Based Approach to *H. pylori* Diagnosis

**DOI:** 10.3390/diagnostics16040521

**Published:** 2026-02-09

**Authors:** Flavia Pigò, Gian Carmine Fernicola, Marinella Lupo, Carlo Ceraso, Libera Esposito, Helga Bertani, Giuseppe Grande, Silvia Cocca, Salvatore Russo, Margherita Marocchi, Maria Marsico, Valentina Boarino, Riccardo Casciola, Rita Conigliaro

**Affiliations:** 1 Gastroenterology and Digestive Endoscopy Unit, Azienda Ospedaliero-Universitaria of Modena, Viale Giardini 1355, 41126 Modena, Italy; 2 Gastroenterology and Digestive Endoscopy Unit, Maresca Hospital, Torre del Greco, 80059 Naples, Italy; 3 Gastroenterology and Digestive Endoscopy Unit, San Paolo Hospital, 80125 Naples, Italy; 4 Digestive Endoscopy Unit, Apuane Hospital, 54100 Massa, Italy

**Keywords:** *Helicobacter pylori*, diagnostic accuracy, gastric biopsy, esophagogastroduodenoscopy

## Abstract

**Background**: Although the incidence of *H. pylori* infection is decreasing globally, it is not completely negligible. Because *H. pylori* infection is associated with various pathologies, ranging from peptic ulcer disease to neoplastic lesions, research into and treatment of *H. pylori* infections remain important. **Objectives**: We aimed to assess the diagnostic performance of gastric juice analysis (Endofaster^®^) for the detection of *H. pylori* in patients undergoing esophagogastroduodenoscopy (EGDS), using conventional histology as the reference standard. Our secondary objectives were to identify the optimal ammonium concentration thresholds for defining positive and negative results and to propose a clinical flowchart to support patient management. **Methods**: The diagnostic accuracy of Endofaster was first analyzed using an unmatched training cohort comprising an equal number of *H. pylori*-positive patients (n = 30) and negative controls (n = 30) who underwent EGDS. The derived thresholds were subsequently evaluated in an independent validation cohort of patients who underwent EGDS with Endofaster. Histological examination is the gold standard for *H. pylori* diagnosis. **Results**: In the training cohort, an ammonium concentration cut-off of 62 ppm/mL yielded a sensitivity of 90% (95% CI: 74–97%) for ruling out *H. pylori* infection. For confirming infection, the optimal cut-off was 100 ppm/mL, corresponding to a specificity of 95% (95% CI: 83–99%). Ammonium values > 62 and <100 ppm/mL were considered indeterminate, suggesting gastric biopsy was required for confirmation. The validation cohort included 196 patients (mean age: 59.9 ± 12.7 years), with a histology-based *H. pylori* prevalence of 19%. In this cohort, Endofaster^®^ demonstrated a sensitivity of 70% (95% CI: 51–85%) and a specificity of 93% (95% CI: 88–97%). Indeterminate results were observed for 29 patients (15%). **Conclusions**: Endofaster^®^ provides a largely reliable diagnosis of *H. pylori* infection during EGDS when a decision-making approach is applied, allowing gastric biopsies to be reserved for indeterminate cases only.

## 1. Introduction

*Helicobacter pylori* (HP) infection causes chronic active gastritis, the initial stage of a disease that leads to the gastric carcinogenic cascade through atrophic chronic gastritis (ACG) and dysplasia. HP is also the main cause of gastric B-cell lymphoma and peptic ulcer disease [1]. Current European guidelines recommend using upper endoscopy with biopsies to detect HP infection in dyspeptic patients with an increased risk of gastric cancer as well as in those with alarm symptoms [2]. However, the incidence of HP infection is decreasing in Western countries. A recent U.S. national study found that the prevalence of HP is below 15% in patients undergoing upper endoscopy [3]. Endoscopy and biopsy sampling are essential for the diagnosis and treatment of certain gastrointestinal diseases. The increasing demand for endoscopic procedures and the associated costs call for rational use of available resources without compromising proper patient management [4]. In recent years, attention to environmental sustainability has also extended to endoscopy, leading to the development of the concept of “Green Endoscopy”, an approach aimed at reducing the ecological footprint of endoscopic procedures through various strategies, such as improving the appropriateness of endoscopic exams and minimizing biopsy use [5]. In this context, Endofaster^®^ (NISO Biomed S.r.l. Turin, Italy) was developed as a device capable of measuring gastric juice in real time by evaluating two parameters: gastric pH, which correlates with the risk of atrophic gastritis (to be confirmed histologically), and ammonium concentration, which is directly proportional to the presence of HP in the stomach [6,7]. Recently, Zullo et al. [8] conducted a review of 11 studies involving Endofaster^®^, concluding that there was a very high negative predictive value for the diagnosis of atrophic gastritis (97%) and HP (96%). The ammonium threshold used in these studies was 60–62 ppm/mL for the identification of HP, but this cut-off may produce false-positive results leading to potentially inappropriate prescription of antibiotic therapy.

A more accurate ammonium threshold for detecting HP during endoscopy would allow the selection of patients who require gastric mucosal biopsy and those unlikely to benefit from biopsy in terms of time and cost.

In this study, we aimed to assess the diagnostic performance of Endofaster^®^ for the detection of *Helicobacter pylori* in patients undergoing EGDS, using conventional histology as the reference standard.

## 2. Materials and Methods

### 2.1. Study Design

We conducted a diagnostic accuracy study aimed at determining ammonium concentration threshold values for the diagnosis and treatment of HP infections using Endofaster^®^ (NISO Biomed, Turin, Italy). The local ethical committee approved this study in 2025 (prot. n. 436/2025).

Thresholds were determined in a cohort of patients consisting of 50% *H. pylori*-positive and 50% *H. pylori*-negative individuals (training set) based on histological examination. The diagnostic threshold value was determined according to the ammonium concentration required to achieve a sensitivity target of 90%, while the treatment threshold was determined based on the ammonium value corresponding to a specificity target of 90%.

The identified thresholds were then tested in a separate patient cohort (validation set) to determine diagnostic accuracy.

### 2.2. Patients

Training set: This set comprised 30 *H. pylori*-positive patients and 30 *H. pylori*-negative patients. These patients were selected from a retrospective cohort (January–June 2023) of individuals who underwent EGDS with Endofaster^®^ and, during the same procedure, gastric mapping biopsies according to the Sydney system [2]. Patients were classified as *H. pylori*-positive or *H. pylori*-negative based on histological results, an approach considered the gold standard.

Validation Set: The ammonium threshold values for the diagnosis and treatment of *H. pylori* infection were tested in a retrospective cohort (July–December 2023) of patients (distinct from the training set) who underwent EGDS with Endofaster^®^ and, during the same procedure, gastric mapping biopsies according to the Sydney system.

Patients were classified as *H. pylori*-positive or -negative based on histologic results, an approach considered the gold standard.

Eligibility criteria: We enrolled adults (>18 years) who were able to provide informed consent. Patients were excluded if blood or food residues were found in the stomach, as such residues may interfere with intragastric ammonium measurement. Patients with a history of gastric surgery were excluded. No proton pump inhibitor (PPI) washout period was required prior to endoscopy.

### 2.3. Procedures

All patients underwent standard white-light upper endoscopy. Biopsy sampling was performed regardless of whether there was suspicion of chronic atrophic gastritis, if gastric intestinal metaplasia was detected during endoscopy, or if the mucosa appeared to be normal. No system for classifying the extent of gastritis was used, and biopsy sampling was performed in accordance with the updated Sydney system [2]. In case of suspicious gastric lesions (e.g., ulcers, neoplasia, or polyps), biopsied material was collected in a separate container for histological analysis. Giemsa staining was used for routine screening for HP; immunochemical staining was used to confirm infection in case of doubt.

This study was conducted using Endofaster^®^, a device available at our center that performs automatic, real-time gastric juice analysis during upper endoscopy. The device is placed between the endoscope and the aspiration system, using 4 mL of gastric juice aspirated at the beginning of the procedure. Detection of HP is based on measurement of ammonium concentrations due to bacterial urease activity within 30–90 s during the endoscopy [6].

### 2.4. Statistical Analysis

The “diagnostic threshold value” is the ammonium concentration below which no further tests are required to rule out HP infection. The “treatment threshold value” is the ammonium concentration above which no further tests are required to confirm the presence of HP. Between these two threshold values lies a diagnostic uncertainty zone, where the test’s accuracy decreases and additional testing is therefore required to confirm or exclude the diagnosis.

The diagnostic and treatment threshold values were determined using the sensitivity/specificity vs. cutoff plots targeting a sensitivity and specificity of at least 90%, respectively, and by defining the grey zone. AUC (area under the curve) and ROC (receiver operating characteristic) curves were estimated to determine the optimal cut-off values. In the validation set, the sensitivity, specificity, and positive and negative predictive values of the Endofaster^®^ device for diagnosing HP infection were calculated using the threshold values identified in the training set [9,10].

For ammonium concentrations below or above the diagnostic threshold values, the following outcomes were considered:

- True positives: *H. pylori* infections determined to be positive via both Endofaster and histological examination.
- True negatives: *H. pylori* infections determined to be negative via both Endofaster and histological examination.
- False negatives: *H. pylori* infections determined to be negative via Endofaster but positive via histological examination.
- False positives: *H. pylori* infections determined to be positive via Endofaster but negative via histological examination.

Continuous data are expressed as means ± standard deviations or medians with 25th–75th percentiles according to a parametric or non-parametric distribution, respectively. Categorical data are expressed as absolute numbers and percentages.

#### Sample Size Considerations

The sample size of the training set was estimated to be sufficient to identify an area under the curve (AUC) ≥ 0.70 as statistically different from 0.50, with 80% power (α = 0.05; two-tailed test). For the validation set, a minimum sample size of 190 patients was considered adequate, based on an expected AUC ≥ 0.70 and a worldwide expected prevalence of 15%, with 80% power (α = 0.05; two-tailed test).

## 3. Results

**Figure 1:**
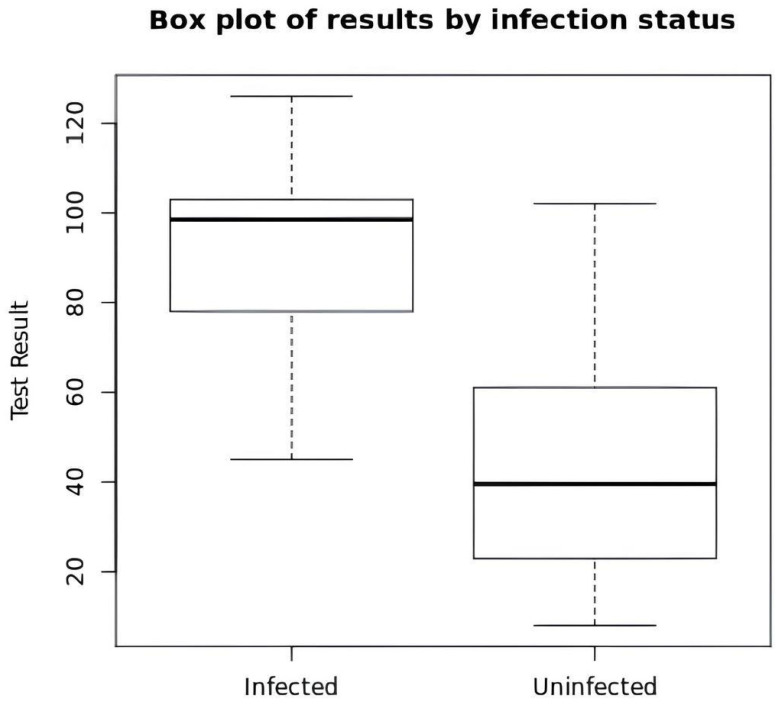
Distribution of ammonium values by *H. pylori* infection status.

Training set: The HP-positive and -negative patient cohorts were both composed of adult subjects (mean age: 56 years); there was a greater prevalence of PPI use and gastrointestinal reflux-related symptoms in the HP-negative patient set. The infected patients had significantly higher ammonium concentrations (median: 98 ppm/mL; 25th–75th percentiles: 45–126) than the non-infected patients (median: 39 ppm/mL; 25th–75th percentiles: 8–102). The baseline characteristics of the patients in the training set are shown in Table 1 and Figure 1.

**Figure 2:**
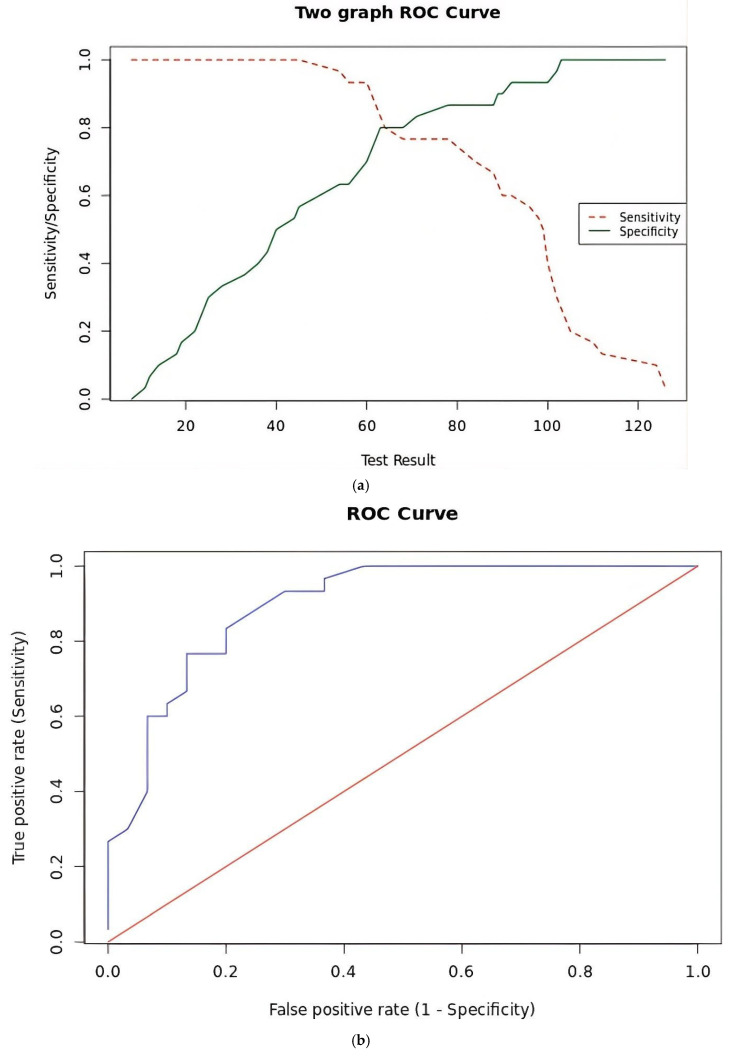
Sensitivity–specificity plot (**a**) and ROC curve (**b**).

For a sensitivity target of 90%, the diagnostic threshold was 62 ppm/mL, yielding a sensitivity of 90% (95% CI: 74–97%), whereas for a specificity target of 95%, the treatment threshold was 100 ppm/mL, corresponding to a specificity of 95% (95% CI: 83–99%). A grey zone was defined between 63 and 99 ppm/mL. The AUC was 90% (95%CI: 82–98%). The sensitivity–specificity plot and ROC curve are shown in Figure 2a,b. These threshold values were subsequently evaluated in an independent validation cohort.

Validation set: The patients in this cohort were mostly adults (mean age: 59.9 ± 12.7 years) with gastrointestinal reflux disease or dyspeptic disorders, and approximately 30% were undergoing PPI therapy. The prevalence of HP infection according to Endofaster^®^ was 30%, whereas that according to histology was 19% (considered the true prevalence). Table 2 shows the baseline characteristics of the validation set.

**Figure 3:**
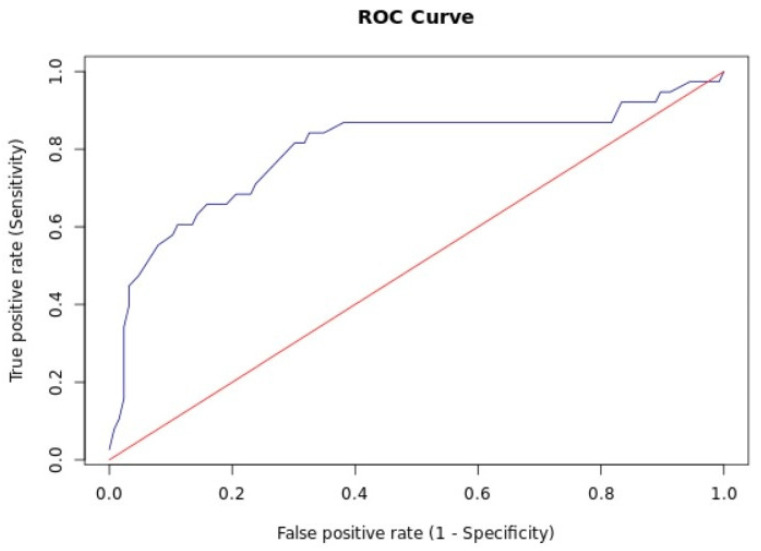
ROC curve for the validation set.

**Figure 4:**
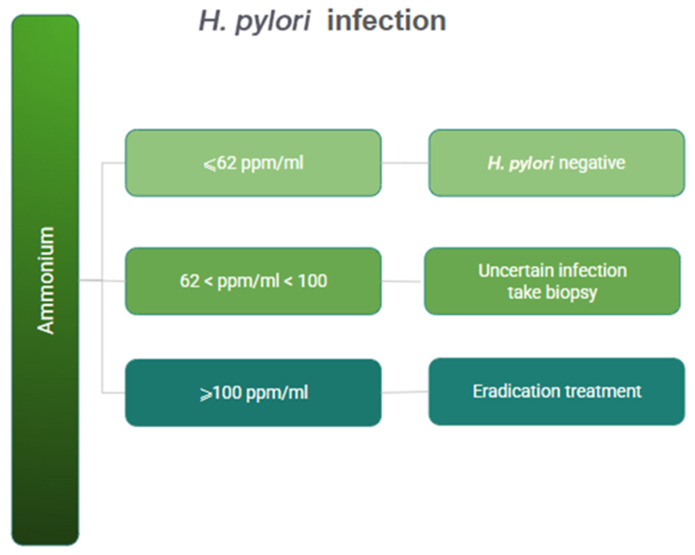
Flowchart for the diagnosis of *H. pylori* with Endofaster.

In the validation set, Endofaster^®^ correctly ruled out HP infection in 70% (95% CI: 51–85) of patients and correctly identified positive patients in 93% of cases (95% CI: 88–96). Fifteen percent of patients in our validation set fell within the grey zone (62 < ammonium < 100) and would therefore have required an additional confirmatory test. Based on the prevalence of HP in our patient cohort, the sensitivity was 70% (95% CI: 51–85%), specificity was 93% (95% CI: 88–97%), the negative predictive value (NPV) was 93% (95% CI: 88–96), while the positive predictive value (PPV) was 71% (95% CI: 56–83) (Table 3 and Table 4). The AUC was 0.79 (95% CI: 0.60–0.89). The ROC curve is displayed in Figure 3. In the subgroup of patients undergoing treatment with proton pump inhibitors, the prevalence of *Helicobacter pylori* infection, as estimated via histology, was 16%, with a lower diagnostic yield compared with the PPI-off group (Table 4). After validating the use of threshold values for the diagnosis of HP, we therefore propose the flowchart in Figure 4.

## 4. Discussion

We assessed the efficacy of Endofaster^®^ in detecting HP infection, defining a “grey zone” (62–100 ppm of gastric-juice ammonium) to optimize sensitivity and specificity. In our validation cohort, Endofaster^®^ achieved a high negative predictive value (NPV: 93%) but a more modest positive predictive value (PPV: 71%), with approximately 15% of patients falling into the grey zone and requiring a confirmatory test before therapy initiation.

These findings are broadly consistent with previous studies with Endofaster that reported variable accuracies in relation to HP prevalence and the adoption of a single threshold (62–67 ppm of ammonium). A multicenter study conducted by Guido Costamagna et al. reported, with a prevalence of infection of 39%, a sensitivity of 90.3%, a specificity of 85.5%, a PPV of 80.2%, and an NPV of 93.1% [11]. More recently, a larger multicenter series demonstrated a prevalence of HP of 17.8%, a sensitivity of 86.3%, a specificity of 83.3%, a PPV of only 52.7%, and an NPV as high as 96.6%, supporting the notion that Endofaster^®^ is more reliable for excluding infection than confirming it in all cases [8]. A dual-cutoff approach (sensitive for exclusion while specific for likely positivity) seems [12] safer than a single threshold, especially when the prevalence of the disease is low.

Other studies have explored the application of a dual-threshold or grey-zone approach for diagnosis of HP with non-invasive tests, such as the 13C-urea breath test and the stool antigen test, defining an indeterminate range where the probability of infection was uncertain [13,14].

In a cohort of 525 patients, with histology used as a reference standard, Endofaster^®^ showed high diagnostic accuracy for *Helicobacter pylori* (sensitivity, 87%; specificity, 84%; and NPV, 97%), unaffected by proton pump inhibitor use or previous eradication therapy [15]. In a study conducted by Vasapolli et al. [16] with a cohort of 161 patients, among patients treated with PPI (n = 67), a reduction in sensitivity (from 97% to 70%), PPV (from 95% to 54%), and accuracy (from 97% to 87%) was observed, whereas specificity and NPV remained almost unchanged (>90%). Our data reveal the test’s capacity to confirm an infection is worse when PPI is ongoing, suggesting a confirmatory test is required.

From a clinical perspective, our data confirm that Endofaster^®^ can be reliably used as a triage tool during gastroscopy: when ammonium levels are below the exclusion cutoff, clinicians may spare patients without additional risk factors or endoscopic lesions from undergoing biopsies, thereby reducing procedural time, cost, and CO_2_ emissions [17]. However, given the significant fraction of patients in the grey zone, a confirmatory test (e.g., histology) remains mandatory before initiating eradication therapy when results are borderline. This is particularly relevant in contexts with low HP prevalence; the grey-zone approach may minimize both undertreatment (false negatives) and overtreatment (false positives), thus optimizing antibiotic stewardship and avoiding unnecessary eradication in uninfected subjects, especially where resources are limited [18].

Since the PPV of Endofaster^®^ is lower than its NPV and the prevalence of HP infection is low, a potentially prudent strategy would be to perform a confirmatory test even in cases with ammonium levels >100 ppm/mL. This approach could reduce unnecessary eradication therapy prescription without significantly increasing the costs associated with a second diagnostic test; additionally, the high NPV appears to remain unaffected by other factors. According to the study by Teriaky et al. [19], the proportion of histological results of gastric biopsies performed during EGDS considered normal was 44% in a cohort of patients with a low incidence of precancerous conditions and HP infection (5% and 7%, respectively). However, the diagnostic yield of biopsies during normal EGDS varies with the incidence of the disease. In countries with a high prevalence of *H. pylori* infection, even in the presence of normal-appearing mucosa, random biopsies increase the likelihood of diagnosing HP infection and precancerous conditions/lesions. A similar study conducted on a different cohort of patients at increased risk of gastric adenocarcinoma and *H. pylori* infection could lead to different conclusions regarding biopsy savings. In a cohort of 585 dyspeptic patients undergoing EGDS in Iran, Esmaeilzadeh et al. found a prevalence of *H. pylori* infection of 80%. The prevalences of intestinal metaplasia, gastric atrophy, and gastric dysplasia were 15.2, 12.6, and 7.9% [20].

The main limitation of our study pertains to the reproducibility of our results. There are potential sources of bias and imprecision. In our study, the characteristics of the population, particularly the prevalence of HP infection, could have influenced the accuracy of the results. The index test itself is subject to analytical imprecision, which can be affected by factors such as true fasting status, concomitant PPI therapy, the presence of other bacterial species, and sample dilution. Similarly, the reference standard, histology, may introduce variability depending on the site of biopsy, the number and size of tissue samples collected, and ongoing patient therapies such as recent antibiotic treatments and PPI use. Moreover, the accuracy of histology also depends on the pathologist’s experience and the density of *H. pylori* colonization of the gastric mucosa [21,22]. A combination of two methods for diagnosing *H. pylori* infection could have improved the overall accuracy. Validation of this flowchart in external cohorts of patients is essential to establish its diagnostic accuracy across a larger number of examinations, particularly in relation to different infection and atrophic gastritis prevalences or PPI use.

Similarly, sensitivity- and specificity-based cutoffs can also be used to define threshold values for the suspicion of atrophic gastritis based on gastric pH, providing an additional opportunity to reduce unnecessary biopsies. Zullo et al. have investigated the correlation between Endofaster^®^-measured gastric juice pH and histological diagnoses, such as atrophic gastritis or intestinal metaplasia [23]. As the use of Endofaster^®^ becomes more widespread in clinical practice, further studies exploring the relationship between real-time gastric juice parameters, endoscopic mucosal features, and histological diagnosis will be of great interest, potentially guiding targeted biopsy and patient management strategies. The adoption and implementation of guidelines that improve the appropriateness of the indication for endoscopic examinations and biopsies during EGDS can rationalize available healthcare resources. In the study by Gibson et al., the number of untargeted gastric biopsies reduced by 36% after a common document for appropriateness of examination indications was adopted [24].

In conclusion, our results support the adoption of a dual-threshold plus grey-zone strategy when employing Endofaster^®^, maximizing its strength as a non-invasive, real-time exclusion test while preserving diagnostic accuracy through confirmatory testing in uncertain cases of HP infection. Future studies should aim to standardize grey-zone limits across different populations and assess the cost-effectiveness of this approach in routine clinical practice.

## Figures and Tables

**Table 1:** Characteristics of the training set.

	*H. pylori* Positive	*H. pylori* Negative
N°	30	30
Age (mean ± SD)	57.5 ± 18.1	55.1 ± 17.9
Sex male n (%)	9 (30%)	15 (50%)
PPI on n (%)	3 (10%)	13 (43%)
Endoscopic diagnosis n (%)		
hiatal hernia	0	3 (10%)
Esophagitis	0	5 (17%)
Gastritis	18 (60%)	5 (17%)
Normal	3 (10%)	2 (7%)
peptic ulcer	3 (10%)	0
Gastroduodenitis	1 (7%)	0
Polyp	1 (3%)	1 (3%)
≥1 diagnosis	3 (10%)	14 (46%)
Gastric pH n (%)		
<3	27 (90%)	22 (73%)
3 ≤ pH < 4.5	0	1 (3%)
≥4.5	3 (10%)	7 (23%)
Ammonium levels (ppm/mL), median (25–75°)	98 (45–126)	39 (8–102)

**Table 2:** Characteristics of the validation set.

	Validation Set
N°	196
Age (mean ± SD)	59.9 ± 12.7
Sex (male) n (%)	82 (42%)
Symptoms n (%)	
GERD	52 (26%)
epigastric pain/dyspepsia	61 (32%)
hereditary gastric neoplasm	6 (3%)
GI bleeding	2 (1%)
anemia/celiac disease suspected	18 (9%)
emesis/dysphagia/thoracic pain	15 (8%)
post-treatment follow-up	22 (11%)
IM/AG/dysplasia/surveillance	10 (5%)
≥1 indications	10 (5%)
PPI on n (%)	61 (32%)
other treatments n (%)	17 (8%)
recent EGDS (within 6 months) n (%)	5 (3%)
known HP infection n (%)	8 (4%)
known MI/AG/dysplasia n (%)	10 (5%)
Endoscopic diagnosis n (%)	
hiatal hernia	31 (16%)
Esophagitis	24 (12%)
Gastritis	77 (39%)
Normal	15 (8%)
peptic ulcer	7 (4%)
Gastroduodenitis	14 (7%)
Polyps	2 (1%)
≥1 diagnosis	26 (13%)
Gastric histology n (%)	
Intestinal metaplasia	10 (5%)
Atrophic Gastritis	10 (5%)
I. Metaplasia + A. Gastritis	2 (1%)
Dysplasia	6 (3%)
*H. pylori* infection n (%)	
Histologic diagnosis	
Overall	37 (19%)
PPI off	27 (20%)
PPI on	10 (16%)
Endofaster diagnosis	
Overall	30 (15%)
PPI off	22 (16%)
PPI on	8 (13%)
Gastric pH n (%)	
<3	144 (73%)
3 ≤ pH < 4.5	13 (7%)
≥4.5	56 (29%)

**Table 3:** Accuracy of Endofaster in the validation set.

	Endofaster			
Histology		Ammonium ≥ 100	62 < Ammonium < 100	Ammonium ≤ 62
	*H. pylori* +ve	21	7	9
	*H. pylori* −ve	9	22	128

**Table 4:** Performance of Endofaster^®^ in diagnosing *Helicobacter pylori* infection according to PPI therapy.

	Overall	No PPI	Ongoing PPI
Sensitivity % (95% CI)	70 (51–85)	90 (68–99)	56 (21–86)
Specificity % (95% CI)	93 (88–97)	98 (93–99)	92 (80–98)
PPV % (95% CI)	71 (56–83)	92 (74–98)	56 (30–80)
NPV % (95% CI)	93 (88–96)	98 (91–99)	92 (84–96)

## Data Availability

Data will be made available upon request.
